# Quantitative Determination of Polyphenols and Flavonoids in *Cistus × incanus* on the Basis of IR, NIR and Raman Spectra

**DOI:** 10.3390/molecules28010161

**Published:** 2022-12-25

**Authors:** Sonia Pielorz, Izabela Fecka, Karolina Bernacka, Sylwester Mazurek

**Affiliations:** 1 Department of Chemistry, University of Wrocław, ul. F. Joliot-Curie 14, 50-383 Wrocław, Poland; 2 Department of Pharmacognosy and Herbal Medicines, Faculty of Pharmacy, Wroclaw Medical University, ul. Borowska 211, 50-556 Wrocław, Poland; 3 Department of Fruit, Vegetable and Plant Nutraceutical Technology, Wrocław University of Environmental and Life Sciences, ul. Chełmońskiego 37, 51-630 Wrocław, Poland

**Keywords:** *Cistus*, polyphenols, flavonoids, FRAP, vibrational spectroscopy, multivariate analysis, principal component analysis, partial least squares regression

## Abstract

*Cistus* is a plant that has been used in natural medicine for hundreds of years; it works primarily as an antioxidant and cleansing agent. *Cistus × incanus* leaves or herb can be an attractive source of polyphenols and flavonoids. The official protocols of active compound analysis relies on the extraction of compounds of interest from plant matter, which makes their determination long and costly. An analysis of plant material in its native state can be performed using vibrational spectroscopy. This paper presents a comparison of Raman spectroscopy, attenuated total reflection in mid-infrared and diffuse reflectance technique in the near-infrared region for the simultaneous quantification of total polyphenols (TPC) and flavonoids (TF) content, as well as the determination of FRAP antioxidant activity of *C. incanus* material. Utilizing vibrational spectra and using partial least squares algorithm, TPC and TF were quantified with the RSEP_VAL_ errors in the 2.7–5.4% range, while FRAP antioxidant activity for validation sets was determined with relative errors ranged from 5.2 to 9.3%. For the analyzed parameters, the lowest errors of predictions were computed for models constructed using Raman data. The developed models allow for fast and precise quantification of the studied active compounds in *C. incanus* material without any chemical sample treatment.

## 1. Introduction

The genus *Cistus* is a plant originating from the *Cistaceae* family, including about 180 species of herbaceous plants and shrubs, showing a tendency to interbreed between species [1,2]. The most numerous group of the *Cistus* species is the subgenus *Leucocistus* [3]. Individual species differ in leaf morphology and the content of active substances [4]. Their flowers are hermaphroditic, actinomorphic and hypogeni. The color of petals varies depending on the species, from white and dark pink to light purple [5,6]. Some species are endemic, others widespread in various areas of the Mediterranean, Iberian Peninsula, Canary Islands, Northwest Africa, Italy, Greece and Turkey, depending on climatic and soil conditions [7]. Already in antiquity, *Cistus* sp. were used for colds, coughs, menstrual problems, rheumatism, diarrhea and as an incense [8]. Currently, it is used in medicine, perfumery, cosmetics and for decorative purposes due to its anti-inflammatory [9], antibacterial [10,11], antiviral [12], antioxidant [13,14], antineoplastic [15] and aromatic properties [16]. Currently, *C. incanus* is popular among dietary supplements with antioxidant and anti-infective activity.

The qualitative composition of the individual *C. incanus* material is most often determined for water or hydroalcoholic extracts [17,18]. The specific biological activity of the plant material results from the variety of active compounds, the content of which depends on a number of factors, including genotype and cultivation conditions. Flavonoids and tannins are the main groups of phenolic compounds found in the *C. incanus* leaves or herb [19]. Flavonoids include derivatives of flavonols (myricetin, quercetin and kaempferol) [20,21] and their glycosides (myricitrin, quercitin, hyperoside, tiliroside and others), proanthocyanidins [22] and hydrolyzed tannins, such as ellagitannins (punicalagin and cistusin)[23]. There are also numerous phenolic acids [14,24], including gallic, protacatechic, p-coumaric, chlorogenic, ellagicacids and others [18,25], determining the pro-health values of plant material. 

Polyphenols, including flavonoids, constitute a large group of phenolic secondary metabolites of plants. They are characterized by a large diversity in terms of structure and biosynthetic pathways. They consist of two aromatic rings connected by a three-carbon bridge (C6-C3-C6), formed by a double bond system between carbon atoms and a carbonyl group [26]. The analysis of phenolic compounds and the study of their functions in plants is still a dynamically developing field of research in sustainable development strategies [27]. 

The qualitative and quantitative determination of phenolic compounds in plant tissues, extracts and the products obtained from them is based mainly on high-performance liquid chromatography and spectrophotometry [28,29]. However, determination of various compounds or parameters of interests requires separate procedures, measuring equipment and chemicals, including solvents, reagents and standards, which makes this process expensive and time-consuming. A particularly useful analytical technique that provides high-quality data without chemically treating the plant material is vibrational spectroscopy. Compared to chromatographic techniques, the main advantage of infrared (IR) and Raman spectroscopy is the ability to analyze the material in its native form, which greatly simplifies and shortens the analysis. Therefore, they are used in various types of qualitative and quantitative analyzes of plant matter. Vibrational spectra enable the studies of solid and liquid tissues, including essential oils, aqueous- and organic extracts, as well as all kinds of products obtained as a result of processing plant material. IR spectroscopy is especially dedicated to the analysis of dried or anhydrous samples. Due to the weak Raman scattering of water molecules, Raman spectroscopy is an excellent technique for the analysis of fresh plant material or water extracts obtained from them. Additionally, the application possibilities of spectroscopic techniques have been significantly expanded by the development of imaging techniques. IR and Raman spectroscopy were used, among others, to determine physicochemical properties of leaves and bark of various poplar species, the content of selected organic compounds, e.g., nutrients, chlorophylls or carotenoids. A very exploited branch of analysis with the help of IR and Raman data is the determination of active compounds in plant material, especially those belonging to polyphenols, including flavonoids, tannins and phenolic glycosides [30,31,32].

In this work, we focused on the construction of regression models enabling simultaneous quantitative analysis of the total content of polyphenols, flavonoids and determination of the FRAP antioxidant activity of dried *C. incanus* plant based on vibrational spectra. Three reflection techniques, namely FT Raman, attenuated total reflection (ATR) in the mid-infrared (MIR) and diffuse-reflection (DRIFTS) in near-infrared (NIR) region were used to register spectral data of chemically non-treated plant material.

## 2. Results and Discussion

Commonly used protocols for the quantification of active compounds require time-consuming extractions from plant material. These procedures assume complete leaching of these substances from the tissue. However, the type of the solvent used and the way of treatment of the material do not guarantee quantitative transfer of the compounds of interest to the solution. A separate issue is the presence of non-leachable substances in plant matrix, the presence of which cannot be detected in the extract, but which are still visible in the spectra of post-extraction residues. Additionally, the extraction of other solvent-soluble substances may hinder analysis of the studied compounds. Therefore, vibrational spectroscopy techniques can give a unique possibility of analysis of particular groups of chemical compounds in complex natural samples in their native state, without any chemical treatment.

### 2.1. Vibrational Spectra of C. incanus Material

**Figure 1:**
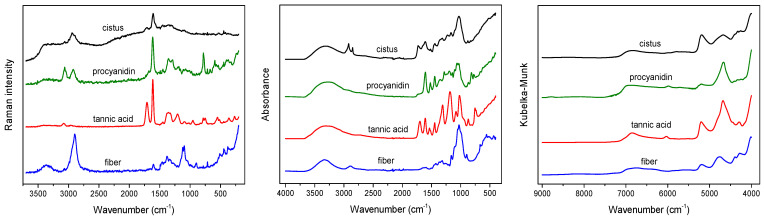
Raman (**left**), ATR (**middle**) and NIR (**right**) spectra of dried *C. incanus* material and selected compounds.

The average vibrational spectra of the studied *C. incanus* samples recorded using the three spectroscopic techniques are shown in Figure 1. In the ATR spectrum of plant material, most characteristic signals can be attributed to the presence of polysaccharides, the structural material of plant tissue. Intense bands visible in the 900–1185 cm^−1^ range with maximum at 1028 cm^−1^ originate from the ν(C–O) and ν(C–O–C) vibrations of carbohydrates’ skeletons and glycosidic linkages [13]. A characteristic massif observed in the 1500–1700 cm^−1^ range results from the overlap of the (C=C) vibrations of polyphenolic compounds and the Amide bands of protein vibrations. In the C–H stretching region, various groups of chemical compounds can contribute. The most pronouncing signals, visible at 2850 and 2919 cm^−1^, originate from the symmetric and asymmetric ν(C–H) vibrations of aliphatic fatty acids. The presence of these compounds in waxes and cutin layers can be additionally identified in ATR spectrum of plant material as the ν(C=O) band of esters present at 1730 cm^−1^ [13,33].

In the case of Raman spectrum of *C. incanus* material, the most intense signal is visible at 1608 cm^−1^, originating from the ν(C=C) stretching vibrations of the aromatic skeleton of phenolic compounds. This peak is associated with much weaker signal at 1705 cm^−1^, which can be assigned to the ν(C=O) vibration of phenolic acids. The bands in the 1250–1490 cm^−1^ range are attributed to the δ(C-OH) phenyl groups deformations and to the δ(CH) and β(CH)vibrations of polysaccharides [33,34]. In the 3100–3500 cm^−1^ frequency range of the Raman spectrum, the stretching vibrations of the hydroxyl groups ν(O–H) can be observed. In the C–H region, a characteristic band with the maximum at 2935 cm^−1^, corresponding to the ν(C–H) stretching vibrations of the aliphatic groups, and much weaker one at 3064 cm^−1^ from aromatic moieties are visible (Figure 1). In spite of the fact that polysaccharides are the dominant structural material, their contributions in Raman spectra of the studied plant tissues are relatively weak.

NIR spectra of *C. incanus* material are less specific compared to ATR and Raman spectra (Figure 1). In the NIR region, the overtones of fundamental vibrations as well as their combinations are present, which usually results in wide bands. The most intense broad signal in the 4900–5400 cm^−1^ range, with the maximum at 5185 cm^−1^ can be linked to the presence of polyphenolic compounds and polysaccharides, while an intense peak with a maximum at 4685 cm^−1^ is commonly associated with the CH group absorption of aromatic species of a phenolic nature [31,33]. Two weak, separate bands, with the maxima at about 4255 and 4322 cm^−1^, originating from δ(C–H) and ν(C–H) combinations of aliphatic fatty acid vibrations, can be detected [32]. Other, very weak bands in the 6100–7500 cm^−1^ range correspond to the presence of carbohydrates, the bands with maxima at 6831 and 6300 cm^−1^ can be assigned to polysaccharides and cellulose, respectively.

### 2.2. Principal Component Analysis (PCA)

Plant material is characterized by high variability of chemical composition. Growing and cultivation conditions, harvest time and other factors can influence the expression of various compounds in the tissues. Similarly, the way the material is stored and processed may affect the content of ingredients of interests. All these factors can be reflected in the vibrational spectra of plant material. 

**Figure 2:**
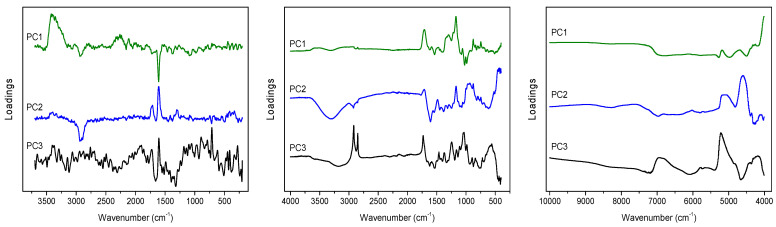
PCA loadings plots obtained on the basis of Raman (**left**), ATR (**middle**) and NIR (**right**) spectra analysis of *C. incanus* material.

PCA on the ATR data matrix revealed that dominant variability in the registered spectra is related to the change in band intensity related to polyphenols content. In the PC1 loadings (Figure 2) the signals at 1170 and 1712 cm^−1^ and about 1300 cm^−1^ can be found, the presence of which will also be detected in the plots of latent variables (LVs) in PLS modeling. Additionally, in this plot the spectral features of polysaccharides appear at 990 and 1024 cm^−1^, which variability is in an opposite direction to signals of polyphenols. In the plot of PC2 loadings (Figure 2) the residual variance of the mentioned 1170 and 1712 cm^−1^ signals is visible, together with variability of the band at 1607 cm^−1^, representing the (C=C) vibrations of aromatic scaffolds, and characteristic broad band from hydroxyl groups vibrations in the 3000–3500 cm^−1^ range. In the PC3 loadings (Figure 2) the variability of lipids can be easily recognized in the form of signals at 1732, 2850 and 2919 cm^−1^, that can be assigned to the νs(C=O), ν_s_(C–H) and ν_as_(C–H) bands, respectively. Interestingly, weak contributions of aliphatic compounds in the (C–H) region are also visible in PC1 and PC2 plots, but with shifted maxima, at 2850 and 2919 cm^−1^ vs. 2852 and 2922 cm^−1^, probably representing variability of various lipid fractions.

**Figure 3:**
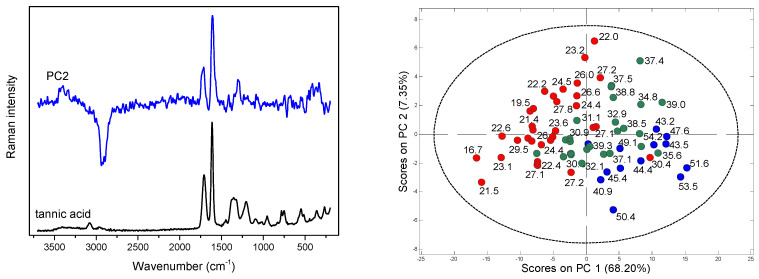
PC2 loadings and Raman spectrum of tannic acid (**left**); scores plots for Raman data with the values of TF (**right**); red points indicate samples containing TF below 30 mg ME/g d.w., green—TF in the 30–40 mg ME/g d.w. range, and blue—TF content above 40 mg ME/g d.w.

Due to the strong fluorescence associated with the Raman spectra of *Cistus* samples, PCA was performed for the baseline corrected data. Spectral variability connected with the presence of polyphenolic compounds is visible in the PC1, PC2 and PC3 loadings plots (Figure 2 and Figure 3) as characteristic signals at 1609 and 1713 cm^−1^ of the stretching (C=C) bands of aromatic scaffolds and (C=O) of the COO^-^ groups of phenolic acids, respectively. 

In addition to these contributions, a strong variability in the (O–H) region is also visible in the PC1 and PC2 loadings, while the variance of the other compounds is less detectable. The scores plots in the PC1/PC2 coordinate system enabled the analysis of the distribution of objects according to the changing content of aromatic scaffolds. This distribution was particularly well ordered in the case of flavonoids content, Figure 3 and Figure S1 in Supplementary Materials.

In the case of NIR data analysis, in the PC1 loadings the bands at 5185 and 4690 cm^−1^ that can be attributed to the variability of polyphenols, and the doublet at 4322 and 4255 cm^−1^ that is characteristic for aliphatic fatty acids, are visible (Figure 2). In the PC2 plot strong contribution at 4600 cm^−1^ and a broad band in the 5000–5200 cm^−1^ range can be found. This latest contribution of aromatic acids origin is also visible in the plot of the PC3 loadings, together with a massif in the 6600–7100 cm^−1^ range representing polysaccharides’ shares (Figure 2).

### 2.3. Quantification of Active Compounds in C. incanus Material

**Figure 4:**
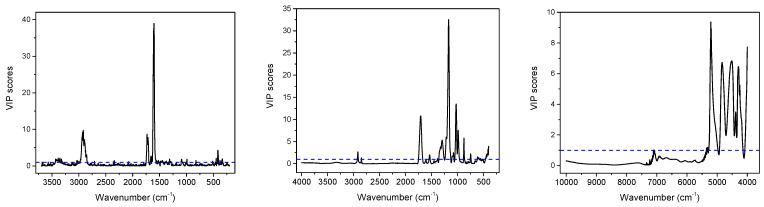
Variable importance in projection (VIP) scores of PLS models for TF obtained on the basis of Raman (**left**), MIR (**middle**) and NIR (**right**) spectra of *C. incanus*.

Partial least squares (PLS) regression models for TPC and TF content determination in dried *C. incanus* samples were developed combining Raman, ATR and NIR spectra of plant material with the results of reference analysis performed for the water-methanol extracts. Establishing the relation between the variability in spectral data and the content of active compounds determined by applying official protocols, allows one to construct chemometric model that can be used for fast quantitative analysis of plant material based on a single vibrational spectrum. Combining the PCA scores plots, a quarter of samples were randomly selected for an external testing procedure and the remaining ones were utilized to develop calibration model. Separate PLS models for polyphenols and flavonoids quantification were constructed for three spectroscopic techniques. For each dataset, applying the VIP scores (Figure 4 and Figure S2 in Supplementary Materials) and selectivity ratio plots (Figure S3 in Supplementary Materials), the spectral ranges were optimized to select those for which spectral variability correlated with the content of analyzed group of compounds. This approach resulted in more reliable models compared to those constructed utilizing entire spectral ranges.

#### 2.3.1. Determination of Total Polyphenols (TPC)

**Figure 5:**
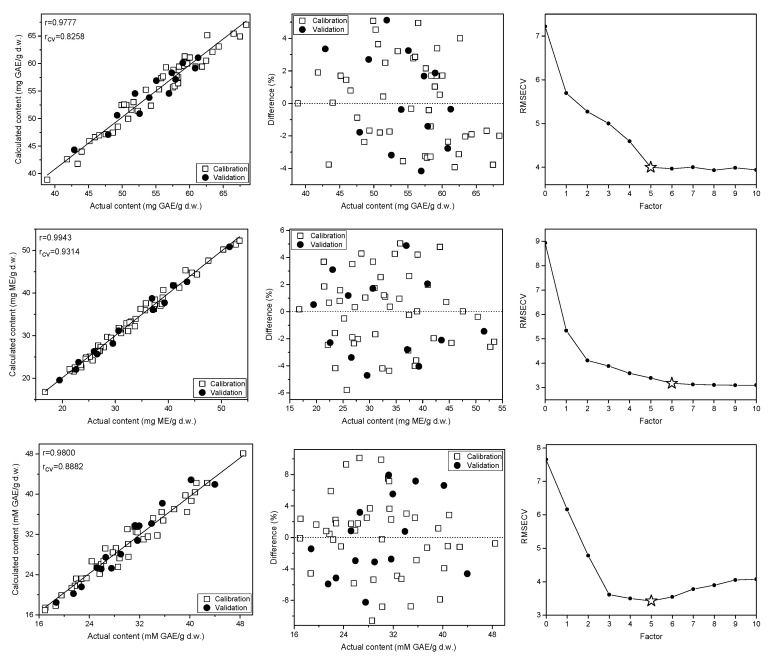
Prediction curves, relative errors and RMSECV plots for the TPC (**top**), TF (**middle**) and FRAP antioxidant activity (**bottom**)determination in the *C. incanus* material on the basis of Raman spectra.

In the studied *C. incanus* material total polyphenols content determined by the Folin-Ciocalteu method ranged from 36.4 to 69.8 mg GAE/g d.w. Four to five PLS factors were necessary to obtain reliable calibration models developed on the basis of Raman, ATR and NIR spectra (Figure 5, Figures S4 and S5 in Supplementary Materials). They were of comparable quality and were characterized by the correlation (r) and cross-validation coefficient (r_CV_) values in the 0.94–0.98 and 0.82–0.84 ranges, respectively. The lowest RSEP errors of TPC determination were found for the model based on Raman spectra and they equaled to 2.7% for calibration and validation sets. In the case of IR techniques, these errors were slightly higher, amounting to 4.1–4.5% and 4.5–4.8%, respectively. The parameters of PLS models for TPC content determination in the studied material are collected in Table 1. The prediction curve, regression residuals and the RMSECV plots obtained for the PLS models are shown in Figure 5, while plots of regression coefficients are presented in Figure S6 in Supplementary Material. The corresponding plots for TPC content modeling utilizing ATR and NIR data together with VIP scores are presented in Figure 4 and Figures S4–S6 in Supplementary Materials.

#### 2.3.2. Determination of Total Flavonoids (TF)

Applying vibrational spectra of the plant material, separate PLS models were constructed to determine TF content. In our samples, concentration of flavonoids found by UV-VIS technique ranged from 16.8 to 53.5 mg ME/g d.w. Interestingly, the correlation between TPC and TF contents was below 0.6, indicating a relatively high proportion of non-flavonoid polyphenols in the studied *C. incanus* samples. The regression models constructed for TF content determination were of better quality than those built for TPC, especially an internal validation of the models gave significantly higher values of the r_CV_ parameter, which reached the level of 0.92–0.97 (Table 1). The PLS models based on Raman and NIR spectra required 6 and 7 factors (Figure 5 and Figure S5 in Supplementary Materials), while only three LVs were necessary to construct robust model using ATR data (Figure S4 in Supplementary Materials). Similar to TPC modeling, the lowest RSEP errors of TF quantification were found for modeling based on Raman spectra. These errors amounted to 2.7 and 2.9% for calibration and testing sets, respectively. In the case of models built using ATR and NIR spectra (Table 1 and Figures S4 and S5 in Supplementary Materials), the RSEP_CAL_ and RSEP_VAL_ values were found in the 3.7–5.4% and 4.4–5.4% range. The plots of prediction curve, regression residuals, the RMSECV parameter and the VIP scores obtained on the basis of Raman spectra modeling are shown in Figure 5, while these plots for TF modeling utilizing ATR and NIR data are presented in Figures S4 and S5 in Supplementary Material. The plots of PLS regression coefficients for the three spectral techniques are shown in Figure S6 in Supplementary Material.

#### 2.3.3. Modeling FRAP Antioxidant Activity

In *Cistus × incanus* leaves material polyphenols and flavonoids are dominant antioxidants, which exhibit high antioxidant activity. In the analyzed samples, the ferric reducing antioxidant power (FRAP) value was found to be in the 16.9–48.5 mM GAE/g d.w. Raman, ATR and NIR spectra were used to construct PLS models to assess the FRAP antioxidant activity of the material. To construct the calibration models, 5 to 7 factors were used. The model developed on the basis of the ATR spectra was characterized by the highest r_CV_ value among the three spectroscopic techniques (Table 1). The relative errors of prediction were in the 4.8–8.6% and 5.2–9.3% range for calibration and validation sets, respectively. Similar to TPC and TF modeling, the lowest RSEP errors were found for models based on Raman data, confirming the highest potential of this spectroscopy technique in determination of parameters related to the content of aromatic species. The prediction curve and regression residuals obtained for PLS model constructed using Raman spectra are shown in Figure 5, and for MIR and NIR data are presented in Figures S4 and S5 in Supplementary Material.

## 3. Materials and Methods

### 3.1. Plant Material

The studied material consisted of 50 commercially available samples of the *Cistus × incanus* L. in the form of dried leaves or herb (shoot tops) from various suppliers, which were purchased in local pharmacies and herbal stores. According to producers’ declarations, plant material came from crops in Albania, Greece and Turkey. For some products, the country of origin was not specified. Each sample was carefully ground and divided into two portions for spectroscopic and reference analysis. Ten additional samples were obtained by mixing different products, spectral analysis were performed on a total of 60 samples. 

### 3.2. Chemicals and Reagents

Folin–Ciocalteu reagent, sodium bicarbonate (Na_2_CO_3_), iron(III) chloride (FeCl_3_) were obtained from Chempur (Piekary Śląskie, Poland). Aluminum (III) chloride (AlCl_3_) was purchased from Fluka (Buchs, Switzerland), and methanol (HPLC grade), and myricitrin (CAS No.: 17912-87-7) from Sigma-Aldrich (St. Louis, MO, USA); gallic acid (CAS No.: 149-91-7) from Extrasynthese (Genay, France).

### 3.3. Reference Analysis

The total phenolics content (TPC) in the studied plant material was determined according to the modified Folin–Ciocalteu procedure [35]. Shortly, 40 µL of Folin–Ciocalteu reagent was diluted with 200 µL of water, then 800 µL of 10% Na_2_CO_3_ solution (*m*/*v*) was added. The samples prepared in this way were incubated in the dark for 30 min and then centrifuged for 7 min. Absorbance was measured at 725 nm for each of the samples applied to the microplate. TPC was determined for the methanol–water extracts, calculated as mg of gallic acid equivalents per gram of dry weight (mg GAE/g d.w.) [36,37]. 

The total flavonoids content (TF) was determined following the European Pharmacopoeia procedure for *Betulae folium* [38], with some modifications. 2% AlCl_3_ solution was diluted with distilled water (50 µL: 50 µL), then, after loading onto a microplate, incubated for 60 min. Absorbance was measured at 420 nm, and TF content in the studied plant material, calculated as mg of myricetin equivalents per gram of dry weight (mg ME/g d.w.), was determined for the methanol–water extracts [36,37].

Antioxidant activity using the FRAP assay was measured according to the Benzie and Strain method [39], with slight modifications. A stock of FRAP reagent was prepared by dissolving 10 mM TPTZ in 40 mM HCl, 20 mM FeCl_3_ and 300 mM acetate buffer (pH 3.6) at the ratio 1:1:10 (*v*/*v*/*v*). Next, 20 µL of each diluted water infusion (1:10, *v*/*v*) or a solution of polyphenol and 200 μL of FRAP reagent were applied to a microplate in triplicate. After 4 min of incubation in darkness at ambient temperature, the absorbance at a wavelength of 593 nm was measured. Results were expressed as mM of gallic acid equivalents per gram of dry weight (mM GAE/g d.w.).

### 3.4. Apparatus

The vibrational spectra of plant material were recorded applying an iS50 FTIR Fourier spectrometer (Thermo Nicolet, Madison, WI, USA). ATR spectra in the 400–4000 cm^−1^ range were recorded using a Golden Gate (Specac, Slough, UK) single-reflection diamond accessory, and NIR spectra in the 3700–10,000 cm^−1^ range were collected utilizing a Collector DRIFTS optical assembly (Thermo Nicolet, Madison, WI, USA). A DTGS detector and KBr beamsplitter were used to collect spectra in MIR range, while in the NIR spectral range an InGaAs detector and CaF_2_ beamsplitter were applied. 96 interferograms were accumulated, Happ-Genzel apodised and Fourier transformed using a zero filling factor of 2. Final MIR and NIR spectra of *C. incanus* material were obtained averaging data from three independent measurements.

Raman spectra were collected using a FT Raman accessory (Thermo Nicolet, Madison, WI, USA) attached to the iS50 unit, equipped with CaF_2_ beamsplitter and an indium-gallium-arsenide (InGaAs) detector. Samples were illuminated by a 1.064 µm Nd:YVO_4_ laser with the power of 150 mW at the sample, a backscattered radiation was collected. The interferograms were averaged over 256 scans, Happ-Genzel apodised and Fourier transformed using a zero-filling factor of 2 to yield spectra in the 100–3700 cm^−1^ range with a resolution of 8 cm^−1^. Raman spectra of powdered *C. incanus* material in the form pellets were collected, during measurements samples were rotated at a constant speed of about 200 rpm. Raman spectra of the studied samples were collected once.

Reference analyses of TPC, TF contents and FRAP activity were performed using a Multiskan GO Microplate Spectrophotometer (Thermo Fisher Scientific, Waltham, MA, USA).

### 3.5. Software and Numerical Data Treatment

Chemometric analysis of spectral data were performed using PLS Toolbox (ver. 6.2, Eigenvector Research, Wenatchee, WA, USA) in a Matlab R2010a (MathWorks, Natwick, MA, USA). TQ Analyst (ver. 7, Nicolet, Madison, WI, USA) software was used to construct initial partial least squares (PLS) models [40,41]. For the PCA and PLS modeling purposes spectral data were mean-centered. In the case of IR data modeling, the spectra were SNV corrected [42]. Separate regression models for TPC and TF determination were constructed for each set of spectra, the variable importance in projection (VIP) score plots were used to optimize spectral regions applied. Validation samples were randomly selected utilizing the samples distribution in PCA scores plots. Internal validation of the PLS models was performed using the leave-*n*-out (*n* = 2) cross-validation. The root-mean-square error of cross-validation (RMSECV) was calculated to select an optimal number of PLS latent variables (LVs). To compare the quality of the developed models the values of relative standard error of prediction (RSEP) were computed, according to the equation:

(1) $$\mathrm{RSEP}\left(\%\right)=\sqrt{\frac{\sum_{i=1}^{n}{\left(C_{i}-{\;C}_{i}^{A}\right)}^{2}}{\sum_{i=1}^{n}C_{i}^{A^{2}}}}\times 100,$$

where $C_{i}^{A}$ is the actual content of the active substance, $C_{i}$—content calculated based on the PLS model, and *n* is the number of samples [43].

## 4. Conclusions

In the course of the research, the usefulness of vibrational spectroscopy for the quantitative determination of active compounds in the dried *Cistus × incanus* leaves was demonstrated. For the first time, a comprehensive performance comparison of different reflection techniques was shown. Utilizing Raman, ATR and NIR spectra the total polyphenols and flavonoids contents were determined using PLS algorithm with the RSEP_VAL_ errors in the 2.7–5.4% range. For the analyzed compounds the smallest errors of predictions, below 3%, were computed for models developed using Raman data. Similarly, in the case of FRAP antioxidant activity based on vibrational spectra, the lowest RSEP errors were found for Raman dataset, despite the lowest S/N ratio of Raman spectra among the three spectroscopic techniques used.

The obtained results indicate that combination of spectral data and multivariate modeling is an effective tool for the simultaneous determination of various compounds in plant material. An undoubted advantage of the developed protocol is the possibility of quantification of the analyzed substances rapidly and directly, without the need for their extraction from the material. The described procedure can therefore be used in the analysis of crucial physical and chemical parameters of raw plant materials in the medical, cosmetic and pharmaceutical industries.

## Figures and Tables

**Table 1:** Calibration parameters of the developed PLS models.

Compound	Parameter		Technique	
		Raman	ATR	NIR
Total polyphenols (36.4–69.8 mg GAE/g d.w.)	r	0.978	0.949	0.935
	r_CV_	0.826	0.831	0.842
	RSEP_CAL_ [%]	2.67	4.13	4.48
	RSEP_VAL_ [%]	2.74	4.50	4.80
	Number of factors	5	4	5
	Spectral range [cm^−1^]	478–563, 719–803, 1543–1672, 2421–3535	500–687, 912–1178, 2378–3652	4972–5318, 5972–5344, 9975–7647
Total flavonoids (16.8–53.5 mg ME/g d.w.)	r	0.994	0.981	0.990
	r_CV_	0.931	0.966	0.915
	RSEP_CAL_ [%]	2.71	5.38	3.65
	RSEP_VAL_ [%]	2.91	5.44	4.36
	Number of factors	6	3	7
	Spectral range [cm^−1^]	410–479, 1056–1135, 1539–1676, 2693–3528	497–688, 908–1405, 2379–3675	5395–7134 7647–8922
FRAP antioxidant activity (16.9–48.5 mM GAE/g d.w.)	r	0.980	0.965	0.947
	r_CV_	0.888	0.942	0.932
	RSEP_CAL_ [%]	4.83	7.24	8.64
	RSEP_VAL_ [%]	5.24	8.37	9.27
	Number of factors	5	6	7
	Spectral range [cm^−1^]	579–767, 1543–1754, 2421–3535	497–688, 907–1487, 1526–1702, 2379–3685	6114–7130, 7638–8743, 4497–5370

r—correlation coefficient, r_CV_—correlation coefficient of cross-validation, _CAL_—calibration set, _VAL_—validation.

## Data Availability

Not applicable.

## Supplementary Materials

The following supporting information can be downloaded at: https://www.mdpi.com/article/10.3390/molecules28010161/s1, Figure S1: PCA scores plots on the basis of ATR and NIR spectra of *C. incanus*; Figure S2: VIP scores for TPC obtained on the basis of Raman, MIR and NIR spectra of *C. incanus*; Figure S3: Selectivity ratio plots for TPC and TF obtained on the basis of Raman, MIR and NIR spectra of *C. incanus*; Figure S4: Prediction curves, relative errors and RMSECV plot for the TPC, TF content and FRAP antioxidant activity in the *C. incanus* material on the basis of ATR spectra; Figure S5: Prediction curves, relative errors and RMSECV plot for the TPC, TF content and FRAP antioxidant activity in the *C. incanus* material on the basis of NIR spectra. Figure S6: PLS regression coefficients for TPC, TF content and FRAP antioxidant activity modeling on the basis of ATR, NIR and Raman spectra.

## Sample Availability

Samples are not available from the authors.
