# Supplementary material for: Quantitative Determination of Polyphenols and Flavonoids in Cistus × incanus on the Basis of IR, NIR and Raman Spectra
Source: Molecules. 2022 Dec 25;28(1):161. doi: 10.3390/molecules28010161 (PMC9822307; doi:10.3390/molecules28010161)
Supplement: Supplementary file 1 [file molecules-28-00161-s001.zip › molecules-2101850-supplementary.pdf]

Quantitative determination of polyphenols and flavonoids in *Cistus* × *incanus* on the basis of IR, NIR and Raman spectra

Sonia Pielorz[1], Izabela Fecka[2], Karolina Bernacka[2,3] and Sylwester Mazurek[1*]

[1] Department of Chemistry, University of Wrocław, ul. F. Joliot-Curie 14, 50-383 Wrocław, Poland

[2] Department of Pharmacognosy and Herbal Medicines, Faculty of Pharmacy, Wroclaw Medical University, ul. Borowska 211, 50-556 Wrocław, Poland

[3] Department of Fruit, Vegetable and Plant Nutraceutical Technology, Wrocław University of Environmental and Life Sciences, ul. Chełmońskiego 37, 51-630 Wrocław, Poland

* Correspondence: sylwester.mazurek@chem.uni.wroc.pl; Tel.: +48 71 375 7307 (S.M.)

SUPPLEMENTARY MATERIALS

Table of contents:

Figure S1. PCA scores plots on the basis of ATR and NIR spectra of *C. incanus*

Figure S2. VIP scores for TPC (top) and FRAP antioxidant activity (bottom) obtained on the basis of Raman, MIR and NIR spectra of *C. incanus*

Figure S3. Selectivity ratio plots for TPC (top), TF (middle) and FRAP antioxidant activity (bottom) obtained on the basis of Raman, MIR and NIR spectra of *C. incanus*

Figure S4. Prediction curves, relative errors and RMSECV plot for the TPC (top), TF content (middle) and FRAP antioxidant activity (bottom) in the *C. incanus* material on the basis of ATR spectra

Figure S5. Prediction curves, relative errors and RMSECV plot for the TPC (top), TF content (middle) and FRAP antioxidant activity (bottom) in the *C. incanus* material on the basis of NIR spectra

Figure S6. Regression coefficient plots for TPC (top), TF content (middle) and FRAP antioxidant activity (bottom) obtained on the basis of Raman, MIR and NIR spectra of C. incanus

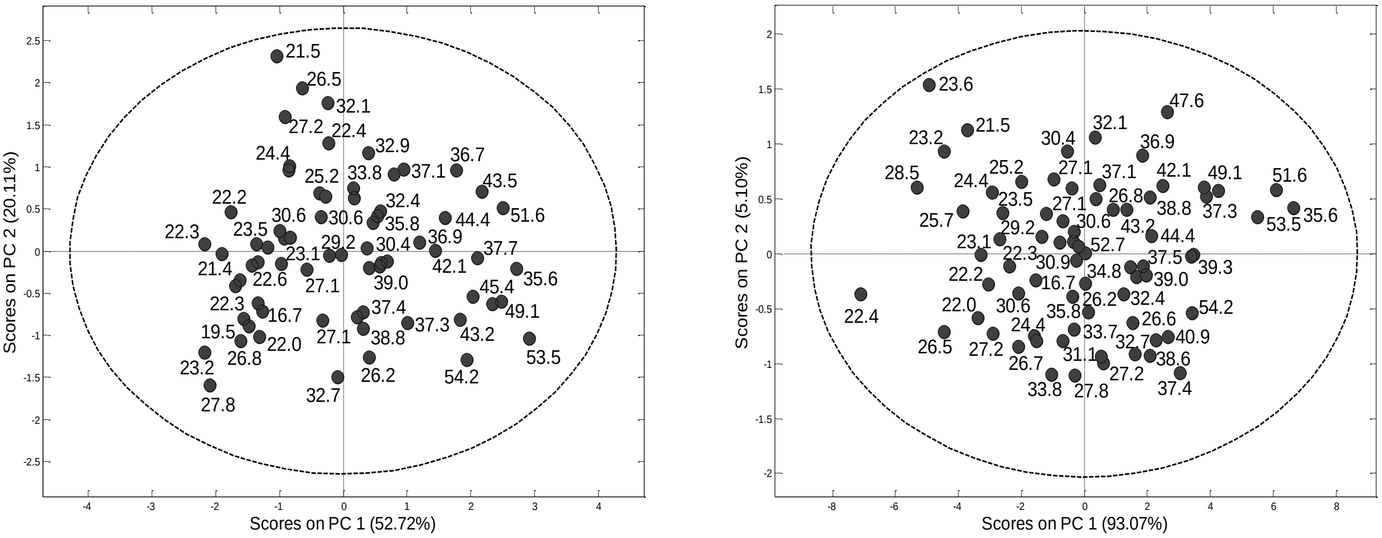

Figure S1. PCA scores plots on the basis of ATR and NIR spectra of *C. incanus*

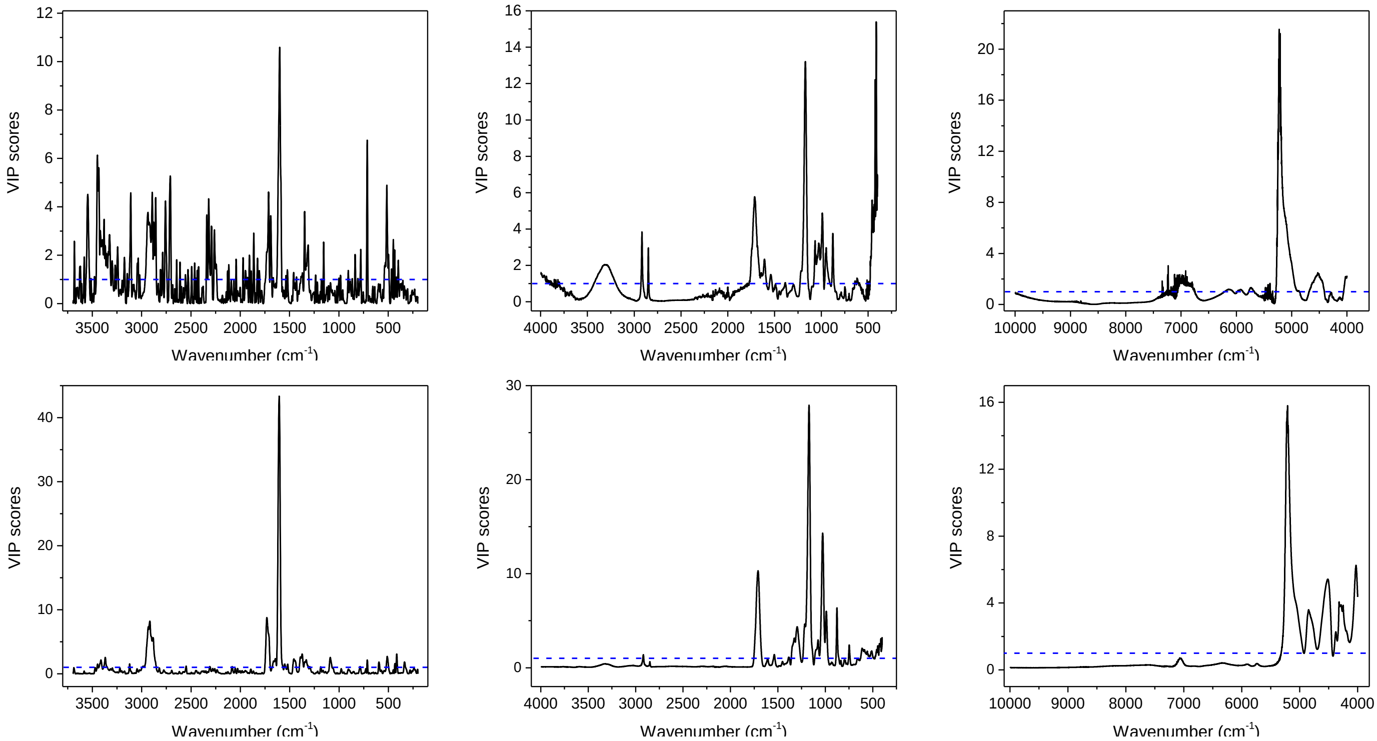

Figure S2. VIP scores for TPC (top) and FRAP antioxidant activity (bottom) obtained on the basis of Raman, MIR and NIR spectra of *C. incanus*

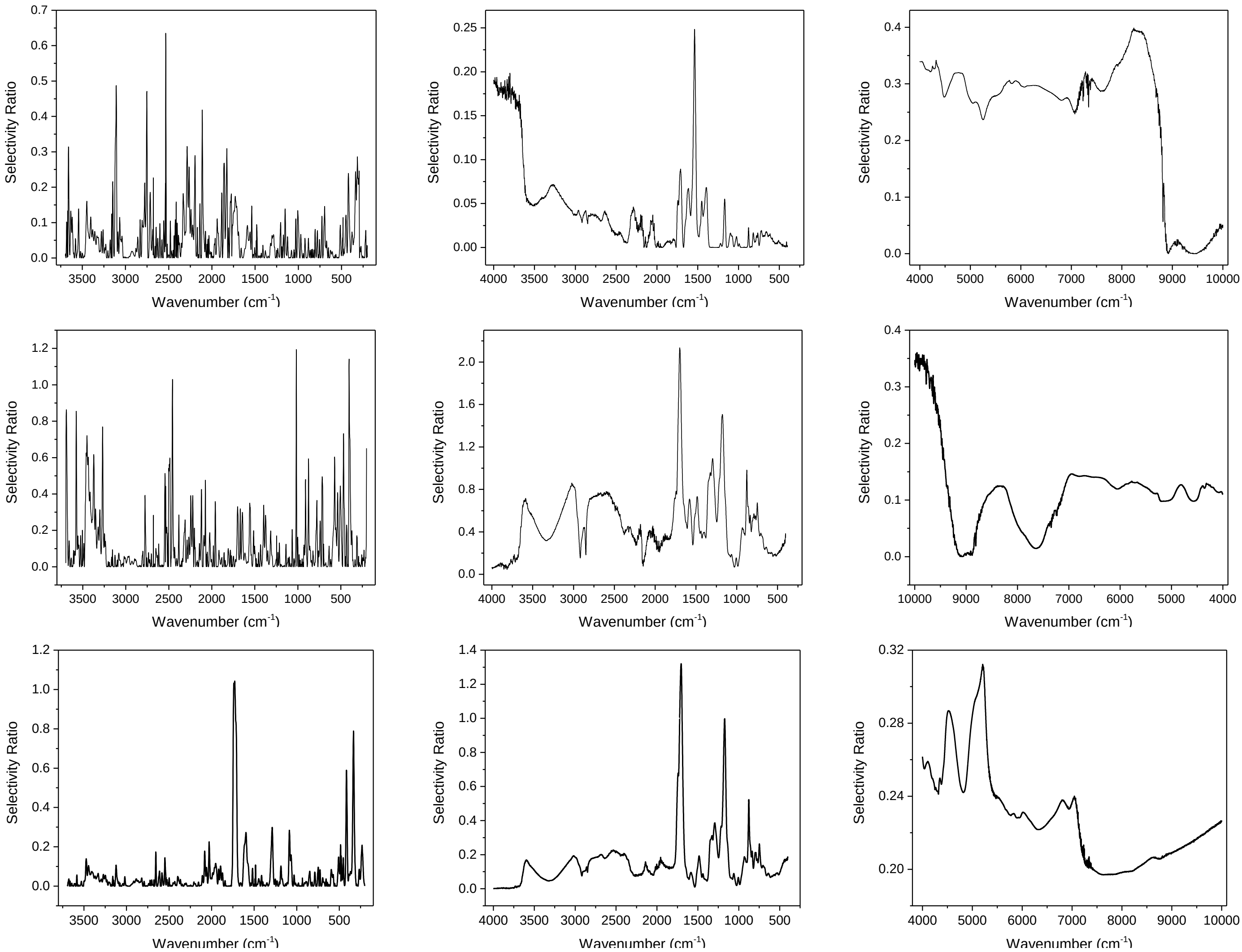

Figure S3. Selectivity ratio plots for TPC (top), TF (middle) and FRAP antioxidant activity (bottom) obtained on the basis of Raman, MIR and NIR spectra of *C. incanus*

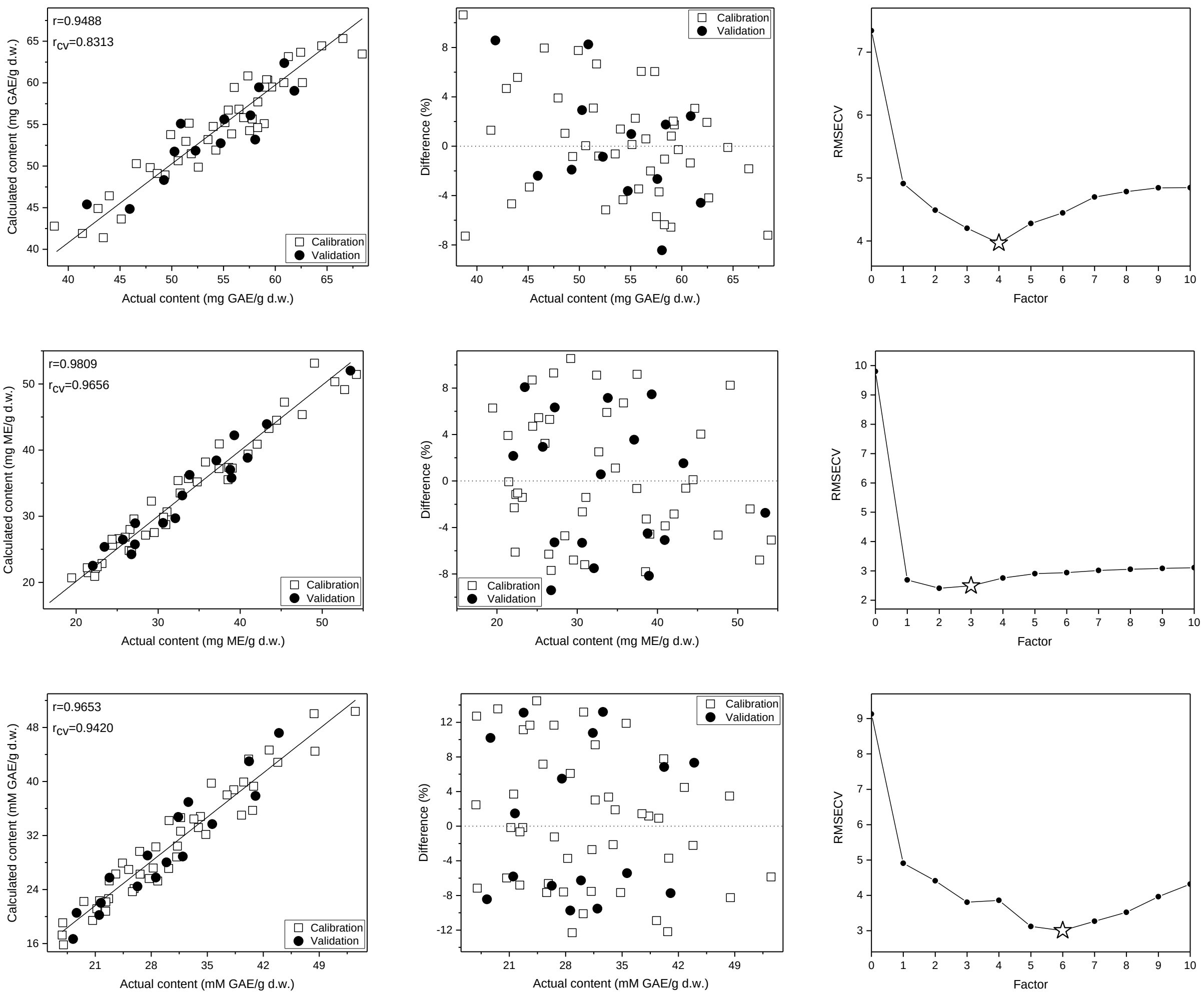

Figure S4. Prediction curves, relative errors and RMSECV plot for the TPC (top), TF content (middle) and FRAP antioxidant activity (bottom) in the *C. incanus* material on the basis of ATR spectra

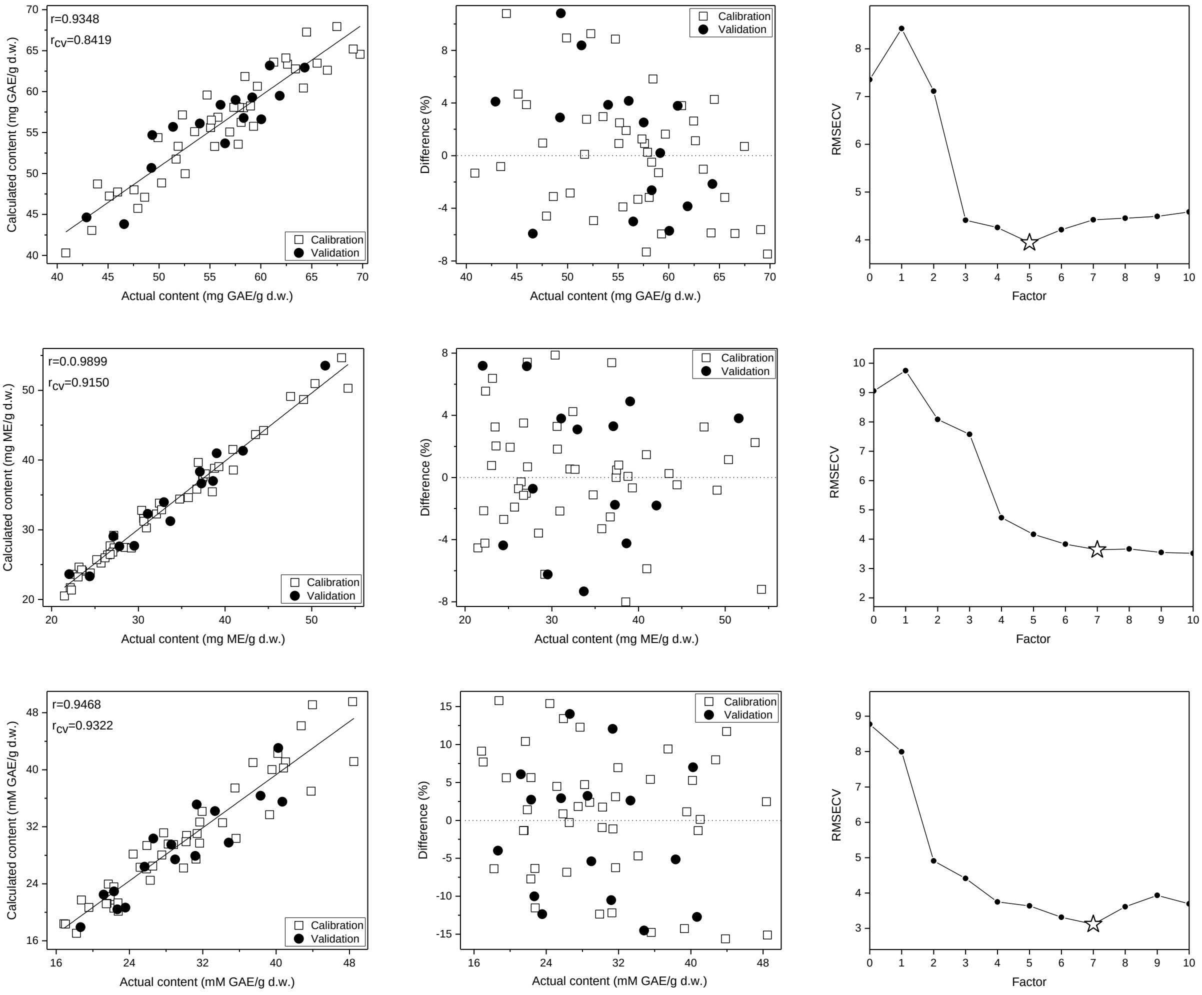

Figure S5. Prediction curves, relative errors and RMSECV plot for the TPC (top), TF content (middle) and FRAP antioxidant activity (bottom) in the *C. incanus* material on the basis of NIR spectra

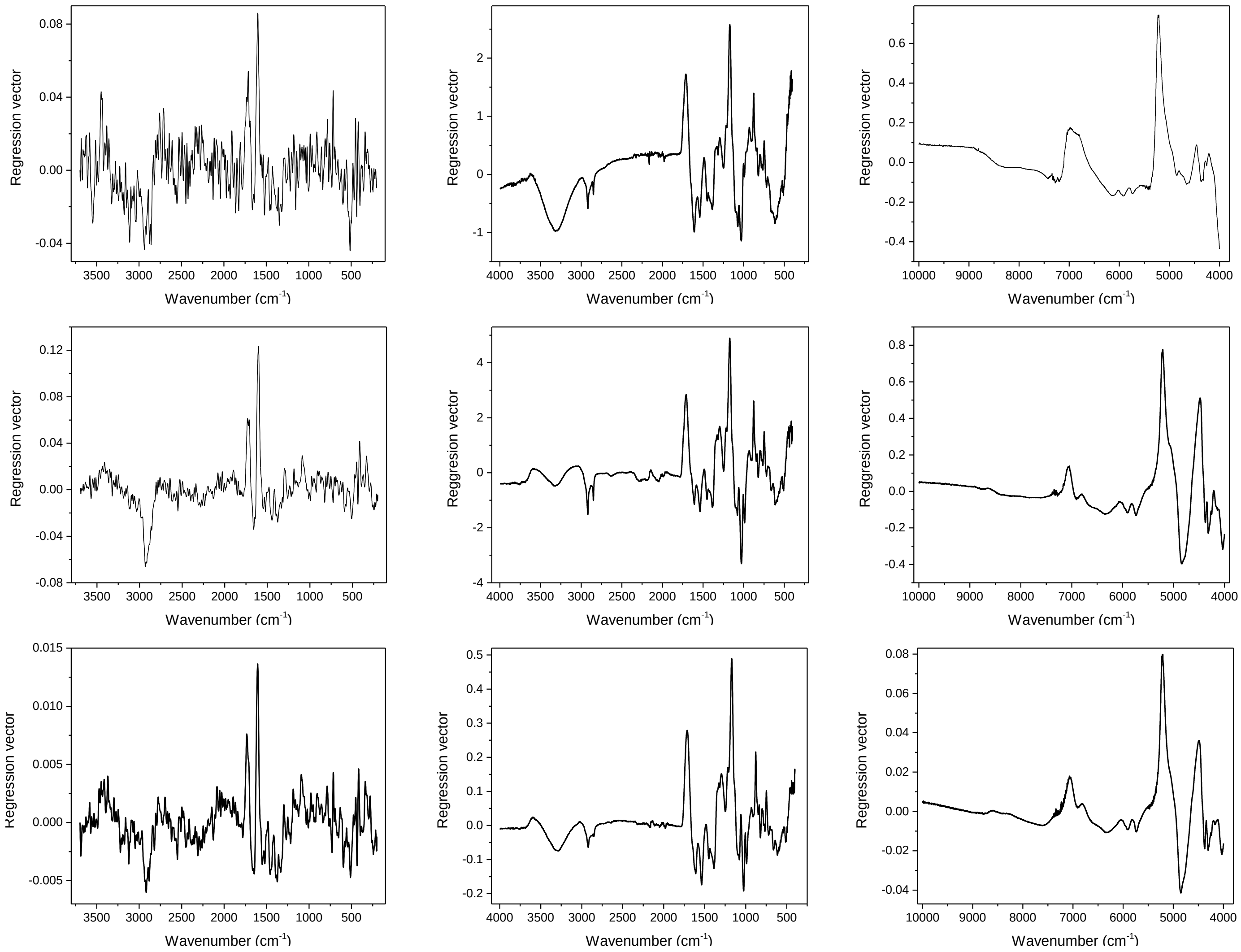

Figure S6. Regression coefficient plots for TPC (top), TF content (middle) and FRAP antioxidant activity (bottom) obtained on the basis of Raman (left panel), MIR (middle panel) and NIR (right panel) spectra of *C. incanus*
